# Immunogenicity and Protective Ability of Genotype I-Based Recombinant Japanese Encephalitis Virus (JEV) with Attenuation Mutations in E Protein against Genotype V JEV

**DOI:** 10.3390/vaccines9101077

**Published:** 2021-09-25

**Authors:** Shigeru Tajima, Satoshi Taniguchi, Eri Nakayama, Takahiro Maeki, Takuya Inagaki, Masayuki Saijo, Chang Kweng Lim

**Affiliations:** Department of Virology I, National Institute of Infectious Diseases, 1-23-1 Toyama, Shinjuku, Tokyo 162-8640, Japan; rei-tani@nih.go.jp (S.T.); nakayama@nih.go.jp (E.N.); tomaeki@nih.go.jp (T.M.); tinagaki@nih.go.jp (T.I.); msaijo@nih.go.jp (M.S.); ck@nih.go.jp (C.K.L.)

**Keywords:** Japanese encephalitis virus, genotype V, reverse genetics, live attenuated vaccine

## Abstract

Genotype V (GV) Japanese encephalitis virus (JEV) has emerged in Korea and China since 2009. Recent findings suggest that current Japanese encephalitis (JE) vaccines may reduce the ability to induce neutralizing antibodies against GV JEV compared to other genotypes. This study sought to produce a novel live attenuated JE vaccine with a high efficacy against GV JEV. Genotype I (GI)-GV intertypic recombinant strain rJEV-E^XZ0934^-M41 (E^XZ0934^), in which the E region of the GI Mie/41/2002 strain was replaced with that of GV strain XZ0934, was introduced with the same 10 attenuation substitutions in the E region found in the live attenuated JE vaccine strain SA 14-14-2 to produce a novel mutant virus rJEV-E^XZ/SA14142m^-M41 (E^XZ/SA14142m^). In addition, another mutant rJEV-E^M41/SA14142m^-M41 (E^M41/SA14142m^), which has the same substitutions in the Mie/41/2002, was also produced. The neuroinvasiveness and neurovirulence of the two mutant viruses were significantly reduced in mice. The mutant viruses induced neutralizing antibodies against GV JEV in mice. The growth of E^XZ/SA14142m^ was lower than that of E^M41/SA14142m^. In mouse challenge tests, a single inoculation with a high dose of the mutants blocked lethal GV JEV infections; however, the protective efficacy of E^XZ/SA14142m^ was weaker than that of E^M41/SA14142m^ in low-dose inoculations. The lower protection potency of E^XZ/SA14142m^ may be ascribed to the reduced growth ability caused by the attenuation mutations.

## 1. Introduction

Japanese encephalitis (JE) is a severe neurological disorder caused by infection with a mosquito-borne arbovirus. Japanese encephalitis virus (JEV) is a critical public health problem in Asian countries. There are an estimated 68,000 cases of JE per year, occurring mainly in China, India, and Southeast Asian countries, resulting in 15,000 fatalities, mostly in children [1,2,3].

JEV belongs to the genus *Flavivirus* in the family *Flaviviridae* and is amplified in a bird/pig-mosquito transmission cycle [4]. The mosquitoes also transmit JEV to humans and horses, which are ‘dead-end’ hosts. JEV has a single-stranded positive-sense RNA genome with a single open reading frame that encodes three structural proteins (C, prM, and E) and seven non-structural proteins (NS1, NS2A, NS2B, NS3, NS4A, NS4B, and NS5). The genome also contains non-coding regions (NCRs) at its 5′- and 3′-terminal ends. JEV is classified into five genotypes (GI, GII, GIII, GIV, and GV) based on genome sequence [5,6]. GIII strains were widely distributed and were most frequently identified in JE endemic areas until the 1990s. However, the major genotype has begun to change from the GIII to GI strain since the early 1990s in most JE endemic areas [7,8,9]. Although the reason for the broad shift from GIII to GI remains unclear, current findings suggest that GI strains circulating in recent years replicate more efficiently in birds and mosquitoes [8,10]. GII strains have been detected in Indonesia, Singapore, Korea, Malaysia, and Australia, whereas GIV strains have been isolated only in Indonesia [11,12].

The first GV JEV (Muar strain) was isolated from a patient with encephalitis in Malaysia in 1952; however, no other GV strain has been identified for >50 years [6]. In 2009, a GV strain was isolated from a *Culex* mosquito pool in China [13]. Subsequently, genomes of GV JEVs were detected in mosquitoes in 2010 and 2012 in Korea [14,15]. Currently, GV is the majority of JEV strains detected in Korea, although GI and GIII strains have also been identified [14,16]. A group in Korea succeeded in isolating GV JEV from a patient with JE [17]. GV JEV endemic areas may spread to other regions in the future. More attention needs to be focused on monitoring the dynamics of circulating JEV strains in JE endemic areas. To understand the characteristics of GV JEV, it is essential to consider the response to emerging GV JEV. In recent years, several groups have attempted to elucidate the growth and pathogenic properties of GV JEV [18,19,20,21,22]. We and the French group showed that GV strains are highly pathogenic in mice compared with the GI and GIII strains, and those structural proteins are involved in viral virulence [18,21]. The growth ability of the GV Muar strain is clearly lower than that of the GI and GIII strains in mouse neuroblastoma cells, and NS2A is associated with the growth characteristics of the Muar strain [22]. Thus, previous reports suggest that GV JEV has different growth and pathogenic characteristics compared to GI and GIII JEV. Our serological analysis showed that the ratio of the neutralization titer against GV Muar to that against GI Mie/41/2002 was less than 1:2 in most JE patient sera in Vietnam and Japan, in which GV JEV has not been identified [23,24]. 

Live attenuated and inactivated JE vaccines currently used are produced from GIII strains [25,26]. A live attenuated JE-yellow fever chimeric vaccine has also been licensed in certain countries, and the vaccine contains prM-E region of an attenuated GIII SA 14-14-2 strain [27]. We previously examined the neutralization efficacy of inactivated GIII Beijing-1-derived JE vaccine against the GV Muar strain in a mouse model; the neutralizing ability of the vaccine against Muar was reduced compared with GI and GIII strains [19]. The Chinese group also indicated that low levels of neutralizing and protective antibodies induced by the SA 14-14-2-derived live attenuated vaccine were observed against the Chinese GV isolate XZ0934 strain [20]. Furthermore, IgGs induced after infection by XZ0934 exhibit poor neutralizing ability against GIII strains [18]. A comparative analysis of E protein amino acid sequences (500 residues) among GI, GIII, and GV JEV strains revealed a higher homology between GI and GIII strains than between GI and GV, or between GIII and GV isolates. There were 40 residues (8%) that differed between the GIII Beijing-1 and GV Muar strains, but fewer than 12 residues (2.4%) and 8 (1.6%) residues were different between the Beijing-1 strain and the GI strains and between the Beijing-1 strain and the GIII strains, respectively [19]. These findings suggest that GV JEV may be distinct from GI and GIII JEV in terms of antigenicity. The lower homology between the GIII vaccine strains and GV isolates may be responsible for the weak efficacy of the vaccines against GV strains.

The SA 14-14-2-based live JE vaccine, which was licensed for commercial application in China in 1989, and is now widely used in China and several other Asian countries, was developed by serial passages of the parent GIII JEV SA 14 strain in mouse brain and primary hamster kidney cells [28]. In the attenuation process, many mutations accumulated in the SA 14-14-2 genome. Ten amino acid differences in the E protein were confirmed between SA 14 and SA 14-14-2, and amino acid residues at these positions were conserved in most JEV isolates, including GV JEV strains (Table S1). Previous reports have demonstrated that mutations in the E protein are mainly responsible for the attenuation of virulence in the SA 14 strain and other virulent strains [28,29,30,31,32,33,34,35,36,37]. These findings raise the possibility that introducing all ten amino acid substitutions found in the E region of SA 14-14-2 strain into that of GV JEV may reduce its virulence and that the attenuated GV JEV could be used as a novel live vaccine that is highly effective against GV infections.

We previously produced an intertypic recombinant JEV strain, rJEV-E^XZ0934^-M41 (E^XZ0934^), in which the E region sequence of the GI Mie/41/2002 strain was replaced with that of the emerging GV strain XZ0934 [19,21]. In this study, we sought to produce a novel live attenuated JE vaccine with high efficacy against GV JEV using the intertypic recombinant strain and by introducing the ten amino acid substitutions in the E region of E^XZ0934^, examined the properties of the strain in vitro and in vivo, and evaluated the efficacy of the recombinant virus as a candidate vaccine against GV JEV infections.

## 2. Materials and Methods

### 2.1. Cell Culture

African green monkey kidney Vero cells (strain 9013), mouse neuroblastoma Neuro-2a cells, and human neuroblastoma IMR-32 cells were cultured at 37 °C in 5% CO_2_ in Eagle’s minimal essential medium (MEM) (Sigma-Aldrich, St. Louis, MO, USA), supplemented with 10% heat-inactivated fetal bovine serum (FBS) (Sigma-Aldrich, St. Louis, MO, USA) and 100 U/mL penicillin-streptomycin (Thermo Fisher Scientific, Waltham, MA, USA).

### 2.2. Viruses

The GI JEV strain Mie/41/2002 (GenBank accession No. AB241119), which was isolated from pig serum in Japan in 2002 [38,39], and the GV JEV strain Muar (GenBank accession no. HM59272), which was isolated from a patient with encephalitis in Malaysia in 1952, were used [19]. The working virus stocks were prepared through amplification in Vero cells.

### 2.3. Recombinant Viruses

**Figure 1:**
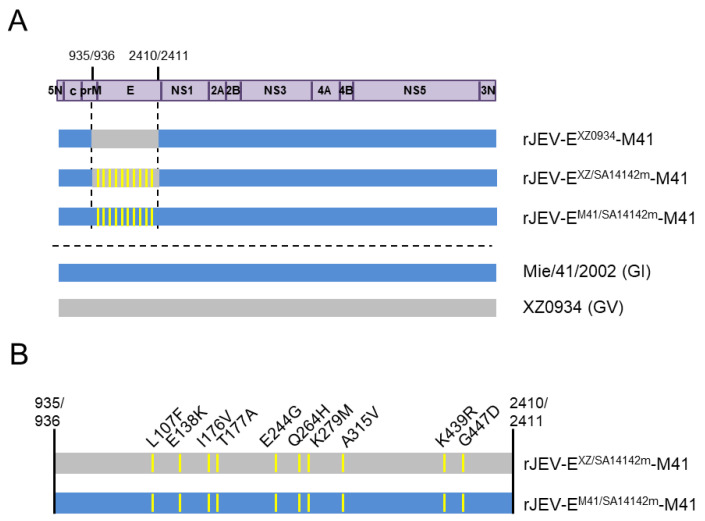
(**A**) Schematic representation of the genomic structure of recombinant JEVs used in this study. Regions derived from GI Mie/41/2002 and GV XZ0934 strains are shown in blue and gray, respectively. Yellow lines indicate point mutation sites. Numbers: nucleotide positions in the Mie/41/2002 genome. (**B**) Enlargement of the E regions of rJEV-E^XZ/SA14142m^-M41 and rJEV-E^M41/SA14142m^-M41 genomes in (**A**). Ten amino acid substitution sites are indicated.

The GI-GV intertypic virus E^XZ0934^ was used [19]. In addition, we constructed additional mutant viruses, rJEV-E^M41/SA14142m^-M41 (E^M41/SA14142m^) and rJEV-E^XZ/SA14142m^-M41 (E^XZ/SA14142m^) in the Mie/41/2002 backbone, as described previously [19,21]. Briefly, the E region of the infectious cDNA clone rJEV(Mie/41/2002)/pMW119 was replaced with the in vitro-synthesized E region DNA of Mie/41/2002 and XZ0934 strains with ten amino acid mutations (Eurofins Genomics, Tokyo, Japan) (Figure 1 and Figure S1). Each synthesized DNA fragment was inserted into the corresponding region using conventional molecular cloning methods and the In-Fusion Cloning system (Takara Bio, Shiga, Japan). The nucleotide sequences of the viral genome regions of the recombinant clones were determined after amplification of the plasmids in *Escherichia coli* STBL2 (Thermo Fisher Scientific, Waltham, MA, USA). Recombinant viruses were recovered by transfecting Vero cells with in vitro-transcribed recombinant viral RNA, as previously described [39]. An aliquot of the culture supernatant of the transfected Vero cells was passaged once in Vero cells, and the culture fluid was used as the recombinant virus solution (v1). The nucleotide sequences of the recombinant viruses were also determined, and no additional nucleotide mutations were detected.

### 2.4. Analysis of Plaque Morphology and Growth Kinetics

Infectious viral titers for each sample were determined using plaque-formation assays. Vero cells (~3 × 10^5^/well) were plated in 12-well culture plates and inoculated with each virus for 1 h at 35–37 °C. Next, a MEM-based overlay medium containing 1% methylcellulose and 2% FBS was added to the wells, and the cells were incubated for 4–5 days at 35–37 °C, after which the cells were fixed using a 10% formalin-PBS solution and stained with methylene blue, as described previously [39].

The in vitro growth ability of the JEV strains was analyzed as described previously [39]. Briefly, cells were cultured in 6-well culture plates and infected with each JEV strain in 3 mL MEM supplemented with 2% FBS (2F/MEM) at a multiplicity of infection (MOI) of 0.05 plaque-forming units (PFU)/cell. Small aliquots (200 μL) of the media were collected at one-day intervals, and infectious viral titers were determined using plaque-formation assays in Vero cells, as described above. Infectious titers of the parental and mutant viruses at 3 days post infection were statistically compared using BellCurve for Excel (Social Survey Research Information, Tokyo, Japan), using student’s *t*-test. The statistical significance was set at *p* < 0.05.

### 2.5. Mouse Challenge Experiment and Sample Collection

Female ddY mice (Japan SLC, Inc., Shizuoka, Japan) were used for mouse challenge tests. For the neuroinvasiveness analysis, groups of mice (3 weeks old, *n* = 10) were inoculated intraperitoneally (i.p.) with 100 μL (1 × 10^5^ PFU) of virus solution diluted in 0.9% NaCl solution. The mice were observed, and the bodyweight of the mice was measured every day for 20 days after inoculation to determine survival rates. Survival curves were compared using BellCurve for Excel and the log-rank (Mantel–Cox) test. Statistical significance was set at *p* < 0.05. The surviving mice were sacrificed, and their sera were collected for further immunological analyses, as described below. For neurovirulence analysis, groups of mice (4 weeks old, *n* = 10 or 5) were inoculated intracerebrally (i.c.) with 30 μL (3 × 10^3^ PFU) of virus solution, and then the mice were observed for 18 days to determine survival rates, as described above.

For protection ability analysis, groups of mice (3 weeks old, *n* = 10) were inoculated i.p. with 100 μL (1 × 10^5^, 1 × 10^4^, or 1 × 10^3^ PFU) of virus (E^M41/SA14142m^ or E^XZ/SA14142m^) solution. In some groups, mice were inoculated again with the recombinant virus solution two weeks after the initial infection. Two or five weeks after the initial inoculation, blood samples of the inoculated mice were collected, and two or three days after collection, the mice were inoculated i.p. with 100 μL (1 × 10^4^ PFU) of virus (Muar) solution. The mice were observed, and the bodyweight of mice was measured every day for 21 days after inoculation to determine survival rates, as described above.

For growth analysis in mice, groups of mice (*n* = 5) were inoculated i.p. with 100 μL (1 × 10^4^ PFU) JEV solution. Serum, brain, and spleen were collected from the mice at two and five days post-infection, and the titer and RNA levels of the infectious virus in the samples were measured, as described below. Tissue weights were determined, and the tissues were homogenized in 500 μL of MEM with 2% FBS, and the homogenate was used for further analyses.

### 2.6. Measurement of Infectious Viral Titer

Viral titers for each sample were determined by plaque-formation assays, as described above, and then statistically compared using BellCurve for Excel, employing the Kruskal-Wallis (Steel-Dwass) test. The statistical significance was set at *p* < 0.05.

### 2.7. Measurement of Viral-Genome Copy Number

The total RNA was extracted from serum and spleen/brain samples using a High Pure Viral RNA Purification Kit (Roche Diagnostics, Mannheim, Germany) and TRIzol LS reagent (Thermo Fisher Scientific, Waltham, MA, USA), respectively. JEV RNA was quantified by RT-qPCR using Fast Virus One-Step Master Mix (Thermo Fisher Scientific, Waltham, MA, USA) as described previously [21]. Briefly, the JEV genome was amplified using primers JE-Multi-f (5′-AGAACGGAAGAYAACCATGACTAAA-3′) and JE-Multi-r (5′-CCGCGTTTCAGCATATTGAT-3′), and JE-Multi (5′-FAM-ACCAGGAGGGCCCGG-MGB-3′) [40]. Genome copy numbers were statistically compared using BellCurve for Excel, using the Kruskal-Wallis (Steel-Dwass) test. The statistical significance was set at *p* < 0.05.

### 2.8. Plaque Reduction Neutralization Test (PRNT)

Neutralizing antibodies against JEV were measured by the PRNT method. Each JEV strain was combined at a 1:1 ratio with 2-fold serial dilutions (1:10 to 1:10,240) of sera from mice infected with JEV and then incubated at 35 °C for 90 min. Vero cell monolayers were inoculated with these mixtures in 12-well plates and incubated at 35 °C for 90 min. Subsequently, overlay medium containing 1% methylcellulose was added, and cells were incubated at 35 °C for 4–5 days. The cells were fixed using a 10% formalin-PBS solution and stained with methylene blue. The PRNT titer (PRNT_50_) was defined as the reciprocal of the highest dilution, resulting in a 50% reduction relative to the mouse serum-free control. PRNT_50_ was statistically compared using BellCurve for Excel, employing the Kruskal-Wallis (Steel-Dwass) test. The statistical significance was set at *p* < 0.05.

### 2.9. Indirect Immunofluorescence Assay (IFA)

Vero cells were infected with the Muar strain at an MOI of 5 and incubated for 24 h. The cells were suspended, and cell smears were prepared on 10-well glass slides and then fixed for 5 min in acetone at room temperature. The cells were exposed to JEV-infected mouse sera, followed by Alexa Fluor 488 goat anti-mouse IgG antibody (Thermo Fisher Scientific, Waltham, MA, USA). The stained cells were visualized by fluorescence microscopy (BZ-X810; Keyence Corp., Osaka, Japan).

## 3. Results

### 3.1. Production of Recombinant JEV Strains with Attenuating E Gene Mutations

Ten amino acid substitutions, L107F, E138K, I176V, T177A, E244G, Q264H, K279M, A315V, K439R, and G447D, were introduced into the E region of E^XZ0934^ to obtain a new recombinant JEV strain, E^XZ/SA14142m^ (Figure 1). Another recombinant virus, E^M41/SA14142m^, which has the same mutations involving the E region of the GI Mie/41/2002 strain, was also produced to compare growth properties, virulence, and neutralization/protection efficacy against GV JEV with E^XZ/SA14142m^.

### 3.2. Growth of E^XZ/SA14142m^ and E^M41/SA14142m^ Strains In Vitro

**Figure 2:**
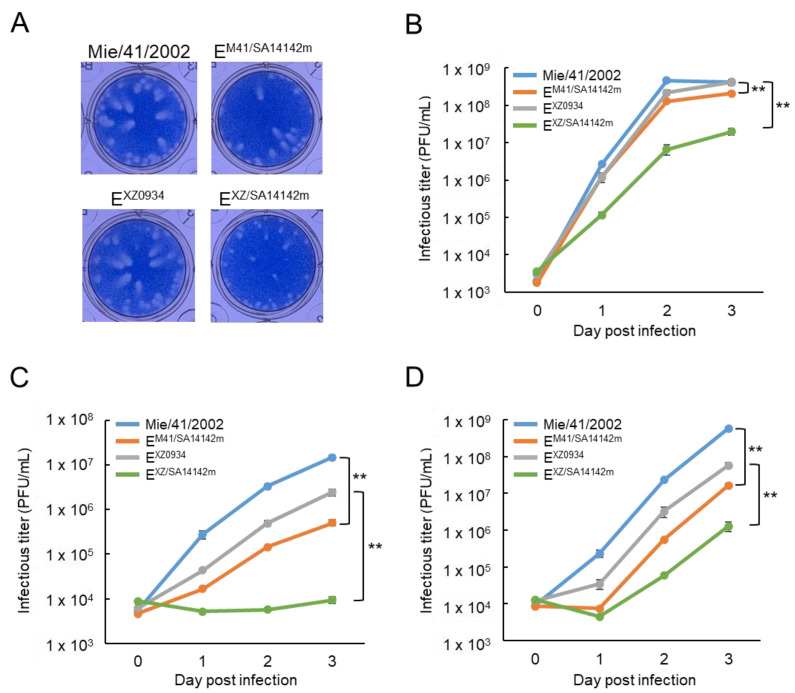
Growth properties of recombinant JEV strains. (**A**) Plaque phenotypes of Mie/41/2002, rJEV-E^M41/SA14142m^-M41 (E^M41/SA14142m^), rJEV-E^XZ0934^-M41 (E^XZ0934^), and rJEV-E^XZ/SA14142m^-M41 (E^XZ/SA14142m^) in Vero cells. (**B**,**C**) Growth curves measured for Mie/41/2002, E^M41/SA14142m^, E^XZ0934^, and E^XZ/SA14142m^ in Vero (**B**), Neuro-2a (**C**), and IMR-32 (**D**) cells. Cells were plated into 6-well culture plates and infected with the JEV strains at a multiplicity of infection (MOI) of 0.1 PFU/cell. Values: means ± standard deviation from three independent inoculations. Significance (parental virus vs. mutant virus, day 3) was analyzed using the student’s *t*-test (** *p* < 0.01).

In Vero cells, plaques formed by E^M41/SA14142m^ were slightly smaller than those formed by the parental Mie/41/2002, while plaques formed by E^XZ/SA14142m^ were considerably smaller than those formed by the parental E^XZ0934^ (Figure 2A). The growth kinetics of E^M41/SA14142m^ was slightly slower than that of the parental Mie/41/2002, whereas E^XZ/SA14142m^ grew obviously slower than the other three strains in Vero cells (Figure 2B). The growth rate of E^M41/SA14142m^ was lower than that of Mie/41/2002 and E^XZ0934^, but E^XZ/SA14142m^ rarely propagated in mouse neuroblastoma Neuro-2a cells (Figure 2C) and human neuroblastoma IMR-32 cells (Figure 2D). These results suggested that the engineered amino acid substitutions decreased the in vitro growth properties of Mie/41/2002 and E^XZ0934^ and that the mutations strongly influenced the growth of E^XZ0934^.

### 3.3. Pathogenicity of E^XZ/SA14142m^ and E^M41/SA14142m^ Strains in Mice

**Figure 3:**
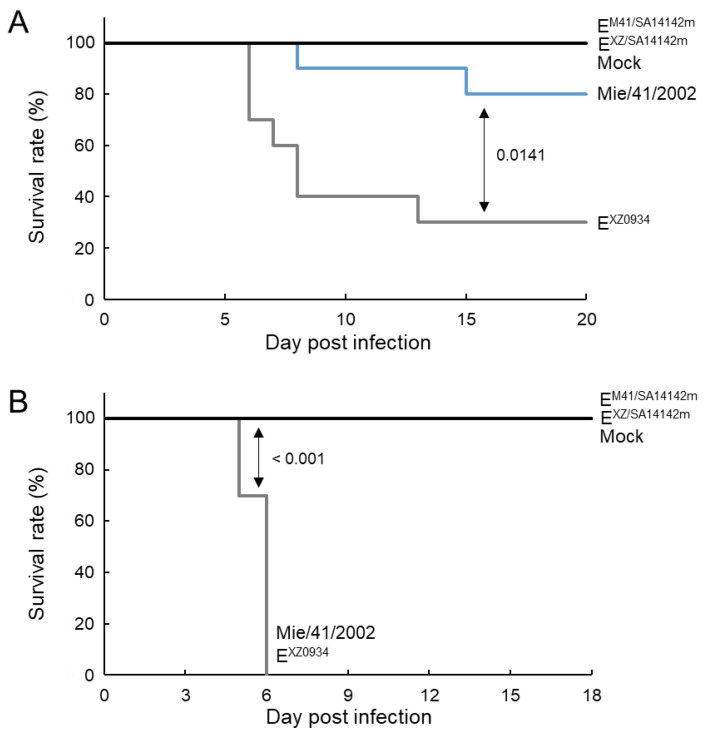
Murine neuroinvasiveness (**A**) and neurovirulence (**B**) of recombinant JEV strains. (**A**) Survival curve of mice inoculated intraperitoneally (i.p.) with 1 × 10^5^ PFU of Mie/41/2002 (*n* = 10), E^M41/SA14142m^ (*n* = 10), E^XZ0934^ (*n* = 10), or E^XZ/SA14142m^ (*n* = 10) and mice mock-inoculated (*n* = 10). (**B**) Survival rate of mice inoculated intracerebrally with 3 × 10^3^ PFU of Mie/41/2002 (*n* = 10), E^M41/SA14142m^ (*n* = 10), E^XZ0934^ (*n* = 10), or E^XZ/SA14142m^ (*n* = 10) and mice mock-inoculated (*n* = 10). Significant *p* values by log-rank test are also indicated.

Mice were inoculated i.p. with Mie/41/2002, E^M41/SA14142m^, E^XZ0934^, and E^XZ/SA14142m^ and observed for 20 days (Figure 3A). Two of ten mice inoculated with Mie/41/2002, and seven of ten mice inoculated with E^XZ0934^ died. None of the mice inoculated with E^M41/SA14142m,^ or E^XZ/SA14142m^ died. An evident loss in body weight was observed in some Mie/41/2002- and E^XZ0934^-inoculated surviving mice, but this was not observed for E^M41/SA14142m^- or E^XZ/SA14142m^-inoculated mice (Figure S2). Mice were inoculated i.c. with the four strains and observed for 18 days (Figure 3B). All ten mice inoculated with Mie/41/2002 and E^XZ0934^ died by day six after inoculation, whereas all ten mice in the E^M41/SA14142m^- and E^XZ/SA14142m^-inoculated groups survived. These results indicated that both the neuroinvasiveness and neurovirulence of Mie/41/2002 and E^XZ0934^ strains were significantly reduced by engineered amino acid substitutions involving the E protein.

### 3.4. Neutralizing Ability of Sera of Mice Inoculated with E^XZ/SA14142m^ and E^M41/SA14142m^ Strains against JEV Strains

**Figure 4:**
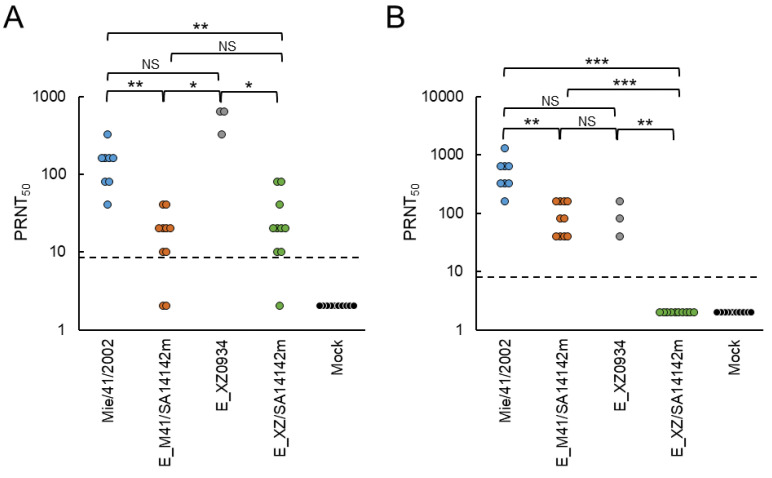
Neutralization activity of serum taken from mice inoculated with recombinant JEV strains. Sera were collected from surviving mice inoculated with Mie/41/2002 (*n* = 8), E^M41/SA14142m^ (E_M41/SA14142m, *n* = 10), E^XZ0934^ (E_XZ0934, *n* = 3), or E^XZ/SA14142m^ (E_XZ/SA14142m, *n* = 10) and mice mock-inoculated (*n* = 10) as shown in Figure 3A, and used for measuring PRNT_50_ against JEV Muar (**A**) and Mie/41/2002 (**B**) strains. Dotted line: detection limit. Significance was analyzed using the Kruskal-Wallis test (* *p* < 0.05, ** *p* < 0.01, *** *p* < 0.001); NS: not statistically significant.

Serum samples were collected from the surviving mice at 20 days post-inoculation in the neuroinvasiveness experiment described in Figure 3A. The samples were provided for PRNT assays against JEV GV Muar, GI Mie/41/2002, recombinant E^XZ0934^, E^XZ/SA14142m^, and E^M41/SA14142m^ (Figure 4 and Table 1). Sera from mice inoculated with E^XZ0934^ showed higher PRNT_50_ (1:320–640) against the Muar strain, and sera from Mie/41/2002-inoculated mice also exhibited titers between 1:40 and 1:320 (Figure 4A). PRNT_50_ in the E^XZ/SA14142m^-inoculated mice group resembled that in the E^M41/SA14142m^-inoculated group, but the titer distributions of the two groups were significantly lower than those of Mie/41/2002- and E^XZ0934^-inoculated groups. Similar results were also observed when E^XZ0934^ was used as the challenge virus for neutralization assays (Table 1). PRNT_50_ against Mie/41/2002 was significantly higher in the Mie/41/2002-inoculated group (1:160–1280) (Figure 4B). Sera from E^M41/SA14142m^- and E^XZ0934^-inoculated mice exhibited titers between 1:40 and 1:160, but no obvious neutralizing activity was detected in E^XZ/SA14142m^-inoculated mice sera (<1:10) against Mie/41/2002. The neutralizing ability of the sera from E^M41/SA14142m^- and E^XZ/SA14142m^-inoculated mice against E^M41/SA14142m^ and E^XZ/SA14142m^ strains, respectively, were also examined, and the titers in the E^M41/SA14142m^ group (1:80–1280) were higher than those in the E^XZ/SA14142m^-inoculated group (<1:10–1:160) (Table 1). Thus, our data indicated that E^XZ/SA14142m^ and E^M41/SA14142m^ could induce neutralizing antibodies against GV JEV in mice. In contrast, the induction ability of E^XZ/SA14142m^ was slightly lower than that of E^M41/SA14142m^.

### 3.5. Growth of E^XZ/SA14142m^ and E^M41/SA14142m^ Strains in Mice

**Figure 5:**
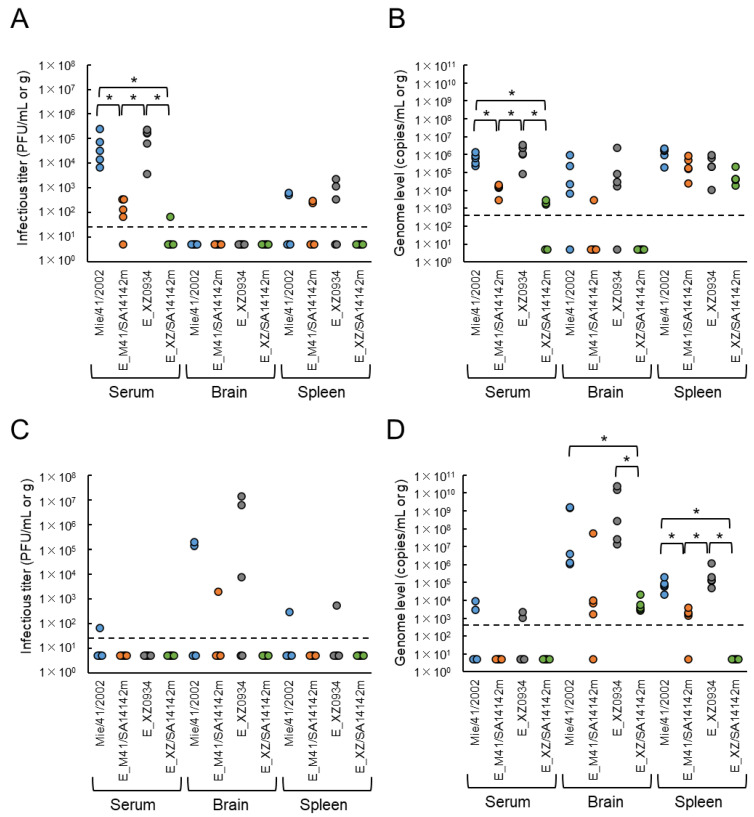
Levels of infectious virus (**A**,**C**) and viral RNA (**B**,**D**) at two (**A**,**B**) and five (**C**,**D**) days after inoculation of JEV-infected mice. Mice inoculated i.p. with 1 × 10^4^ PFU of Mie/41/2002 (*n* = 5), E^M41/SA14142m^ (E_M41/SA14142m, *n* = 5), E^XZ0934^ (E_XZ0934, *n* = 5), or E^XZ/SA14142m^ (E_XZ/SA14142m, *n* = 5) were euthanized at two or five days after inoculation, and serum, brain, and spleen samples were collected. Sera and tissue homogenates were used to quantify the infectious virus titer (PFU/mL or g) (**A**,**C**) and viral genome (genome copies/mL or g) (**B**,**D**). Dotted line: detection limit. Significance was analyzed using the Kruskal-Wallis test (* 0.01 < *p* < 0.05).

Infectious virus and viral RNA levels in mice inoculated with the recombinant strains by i.p. were investigated (Figure 5). Two days after inoculation, no infectious virus was seen in the brain in any four groups, whereas infectious viruses were detected in serum and spleen samples in most groups (Figure 5A). High levels of viremia were observed in sera from mice inoculated with the Mie/41/2002 and E^XZ0934^ strains. Viremia was also observed in four of five E^M41/SA14142m^-inoculated mice and one of five E^XZ/SA14142m^-inoculated mice. Infectious viruses were detected in spleen samples of some mice inoculated with Mie/41/2002, E^XZ0934^, and E^M41/SA14142m^ strains, but not in those inoculated with E^XZ/SA14142m^. Levels of viral RNA in serum and brain samples were also significantly higher in Mie/41/2002- and E^XZ0934^-inoculated mice than in E^M41/SA14142m^- and E^XZ/SA14142m^-inoculated animals (Figure 5B). Five days after inoculation, infectious viruses were not seen in most serum and spleen samples in all four groups. However, a high titer of infectious virus was detected in the brain in two of five Mie/41/2002- and three E^XZ0934^-inoculated groups (Figure 5C). No infectious virus was detected in the brains of all five E^X^^Z/SA14142m^-infected mice. Levels of viral RNA in the brain and spleen samples were also significantly higher in the Mie/41/2002- and E^XZ0934^-inoculated groups than in E^M41/SA14142m^- and E^XZ/SA14142m^-inoculated animals (Figure 5D). These data indicated that the mutant viruses E^M41/SA14142m^ and E^XZ/SA14142m^ had a lower growth ability than the parental Mie/41/2002 and E^XZ0934^ viruses in mice and E^XZ/SA14142m^ exhibited the lowest replication capacity of the four strains.

### 3.6. Protective Efficacy of E^XZ/SA14142m^ and E^M41.SA14142m^ Strains against GV Muar in Mice

**Figure 6:**
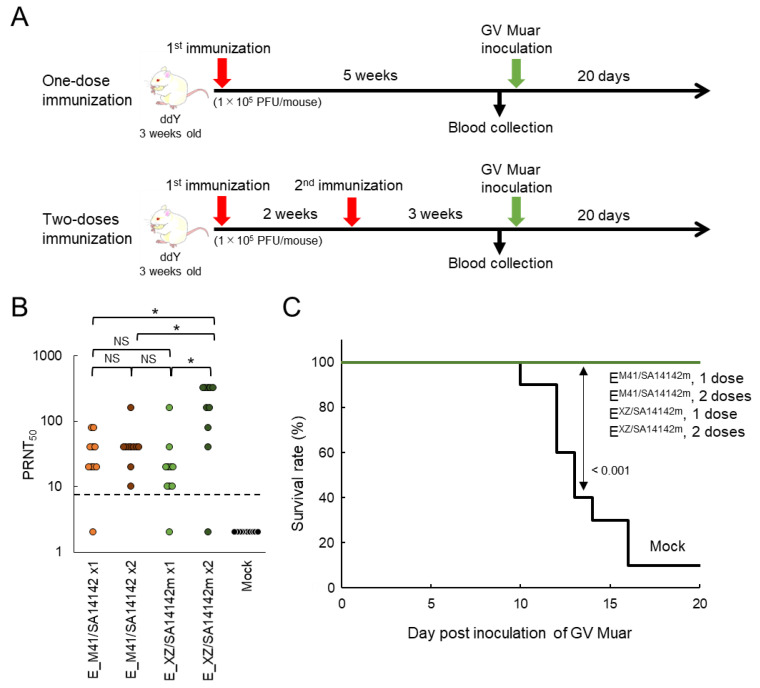
Protective capacity of high-titer recombinant JEV strains against GV Muar strain in mice. (**A**) Experimental timeline. For single-dose immunization groups, mice were inoculated i.p. with 1 × 10^5^ PFU of E^M41/SA14142m^ (*n* = 10) or E^XZ/SA14142m^ (*n* = 10), or mock-inoculated (*n* = 10). Double-dose immunization group mice were inoculated i.p. with 1 × 10^5^ PFU of E^M41/SA14142m^ (*n* = 10) or E^XZ/SA14142m^ (*n* = 10) and were immunized again two weeks after the first immunization. At five weeks after the initial immunization, mice were bled to determine serum PRNT_50_ and then inoculated i.p. with 1 × 10^4^ PFU of Muar strain and observed for 20 days. (**B**) Neutralization activity of serum taken from immunized mice against GV Muar. Dotted line: detection limit. Significance was analyzed using the Kruskal-Wallis test (* 0.01 < *p* < 0.05); NS: not statistically significant. (**C**) Survival curve of immunized mice after challenge with GV Muar strain. Significant *p* values by log-rank test are also indicated.

Our results indicated that the mutant viruses E^M41/SA14142m^ and E^XZ/SA14142m^ display little or no virulence in mice, implying that these strains can be used as live attenuated vaccines against JEV infections. To investigate this possibility, mice inoculated once or twice with 1 × 10^5^ PFU of E^M41/SA14142m^ and E^XZ/SA14142m^ were inoculated i.p. with the GV Muar strain (Figure 6A). Before the Muar strain challenge, blood samples were collected from the mice, and PRNT_50_ against Muar was measured (Figure 6B and Table 2). Neutralizing antibodies against Muar were induced in most mice inoculated with the strains. However, PRNT_50_ was significantly higher in mice inoculated twice with E^XZ/SA14142m^. No obvious differences were observed in terms of neutralizing titers among the other three groups. No neutralization abilities (>1:10) were detected in three mouse sera, but antibodies against Muar were detected in the sera of the mice by IFAs (Table 2). All mice immunized with one or two doses of E^M41/SA14142m^ or E^XZSA14142m^ survived, while nine of ten mock-immunized mice died by day 16 after Muar challenge (Figure 6C). No evident loss in body weight was observed in the mutant-inoculated mouse groups (Figure S3).

**Figure 7:**
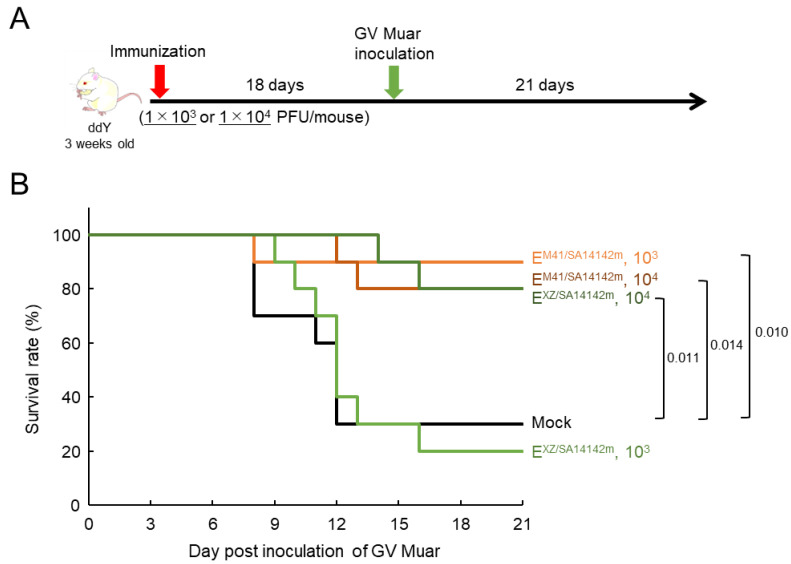
Protective ability of low-titer recombinant JEV strains against GV Muar strain in mice. (**A**) Experimental timeline. Mice were inoculated i.p. with 1 × 10^3^ PFU or 1 × 10^4^ PFU of E^M41/SA14142m^ (*n* = 10) or E^XZ/SA14142m^ (*n* = 10), or mock-inoculated (*n* = 10). At 18 days after immunization, mice were inoculated i.p. with 1 × 10^4^ PFU of Muar strain and observed for 21 days. (**B**) Survival curve of immunized mice after challenge with GV Muar strain. Significant *p* values by log-rank test are also indicated.

Next, mice were inoculated with lower doses (1 × 10^3^ and 1 × 10^4^ PFU) of E^M41/SA14142m^ or E^XZ/SA14142m^, and at 18 days after immunization, the mice were challenged i.p. with Muar (Figure 7A). One and two of ten mice immunized with 1 × 10^3^ and 1 × 10^4^ PFU of E^M41/SA14142m^, respectively, died, whereas eight and two of ten mice immunized with 1 × 10^3^ and 1 × 10^4^ PFU of E^XZ/SA14142m^, respectively, died (Figure 7B). Seven of ten mock-immunized mice died. Some mice with rapid and severe body weight loss survived, regardless of the virus used for immunization (Figure S4). This result suggested that the protective potential of E^XZ/SA14142m^ against Muar may be weaker than that of E^M41/SA14142m^.

## 4. Discussion

In this study, we sought to develop a recombinant live attenuated JE vaccine that is effective against GV JEV infection using a reverse genetics system. In our previous study, we generated a recombinant GI-GV intertypic JEV E^XZ0934^, encoding the E protein of a recent GV isolate, XZ0934, using the GI strain Mie/41/2002 backbone [19]. We used E^XZ0934^ to generate a novel mutant virus E^XZ/SA14142m^. Ten amino acid substitutions related to the attenuation of the GIII JEV strain SA 14 were introduced in the E region of E^XZ/SA14142m^. In addition, we also produced a recombinant mutant virus, E^M41/SA14142m^, that was introduced to the same mutations in the E region of GI Mie/41/2002. The in vitro and in vivo properties of the mutant viruses were examined, and their ability to serve as a live attenuated vaccine against the GV JEV infection was evaluated. Previously, we generated a recombinant intertypic virus, rJEV-5NCME^XZ0934^-M41, in which the 5′NCR-C-prM-E region was replaced with that of XZ0934 in the Mie/41/2002 backbone [21]. However, the prM protein is also involved in the high virulence of GV JEV; therefore, E^XZ0934^ was selected for this study.

Previous reports have shown that the introduction of multiple mutations found in the E region of SA 14-14-2 reduces the proliferative capacity of GI and GIII JEV [33,34,35,41]. Although E^M41/SA14142m^ and E^XZ/SA14142m^ also exhibited reduced growth capability compared with the parental viruses in Vero cells, the mutants maintained a peak infectious titer of approximately 10^7^ PFU/mL (Figure 2B), indicating that viral replication was not critically impaired. However, the mutants presented marked decreases in proliferative ability in mouse neuroblastoma Neuro-2a and human neuroblastoma IMR-32 cells (Figure 2C,D). Moreover, no evident increase in infectious virus levels was observed in the culture supernatant of Neuro-2a cells inoculated with E^XZ/SA14142m^ (Figure 2C). The growth rate of E^XZ0934^ was also lower than that of Mie/41/2002, and these intertypic viruses may be more sensitive to additional mutations than Mie/41/2002 in virus replication.

The mutants E^XZ/SA14142m^ and E^M41/SA14142m^ showed lower neuroinvasiveness than the parental strains in mice (Figure 3A). Infectious virus levels and genomic RNA levels in peripheral tissues and the brain were also lower in mice infected with the mutant viruses than in those infected with the parental viruses (Figure 5), suggesting that the low neuroinvasiveness of the mutants is due to the low proliferative potential of the mutants. In the neurovirulence analysis, all mice inoculated with the parental strains died within 6 days. In contrast, all mice inoculated with the mutant viruses survived until the end of the observation period without any symptoms (Figure 3B). This result indicated that the mutations substantially reduced the replicative ability and cytotoxicity of the parental viruses in central neurons.

In neutralization analysis using sera from surviving mice in Figure 3A, PRNT_50_ in sera inoculated with E^XZ/SA14142m^ or E^M41/SA14142m^ against GV Muar was lower than those in sera inoculated with E^XZ0934^ or Mie/41/2002 (Figure 4 and Table 1). These results could be attributed to the low proliferative potential of mutant viruses in mice. There was no significant difference in PRNT_50_ against Muar between E^M41/SA14142m^-inoculated mouse sera (<1:10–40, median 1:20) and E^XZ/SA14142m^-inoculated mouse sera (<1:10–80, median 1:80). PRNT_50_ against Mie/41/2002 was 1:40–160 (median 1:80) in E^M41/SA14142m^-inoculated mouse sera, whereas they were all <1:10 in E^XZ/SA14142m^-inoculated mouse sera. Furthermore, PRNT_50_ of E^M41/SA14142m^-inoculated serum against E^M41/SA14142m^ was 1:80–1280 (median 1:320), whereas that of E^XZ/SA14142m^-inoculated serum against E^XZ/SA14142m^ was <1:10–160 (median 1:30) (Table 1). These results raise the possibility that E^XZ/SA14142m^ may have a weaker potential to induce neutralizing antibodies against JEV than E^M41/SA14142m^. The low growth capacity of E^XZ/SA14142m^ may be associated with the low immunogenicity of the mutant virus in mice. The mutant viruses were used for immunization twice; PRNT_50_ against Muar was higher in E^XZ/SA14142m^-inoculated mouse sera (<1:10–320, median 1:320) than in E^M41/SA14142m^-inoculated mouse sera (1:10–160, median 1:40) (Table 2, Figure 6B), implying that the booster effect of E^XZ/SA14142m^ was higher than that of E^M41/SA14142m^.

E^XZ0934^-inoculated mouse sera had a higher neutralizing titer against Muar than Mie/41/2002-inoculated sera, while Mie/41/2002-inoculated mouse sera had higher neutralizing titer against Mie/41/2002 than Muar-inoculated sera. These results are consistent with our previous findings that GI and GV JEV can be separated into antigenically distinct groups [19,24]. PRNT_50_ of E^XZ0934^-inoculated mouse sera against E^XZ0934^ was identical to that against Muar (Table 1), whereas PRNT_50_ of Mie/41/2002-infected and E^M41/SA14142m^-infected mouse sera against E^XZ0934^ appeared to be lower than those against Muar (Table 1). There is a difference of 6 amino acid residues in the E protein between Muar and XZ0934 (Figure S5), and the differences in E protein could influence the reactivity of sera with GV E proteins.

Our results demonstrated that a single inoculation with 1 × 10^5^ PFU of E^XZ/SA14142m^ or E^M41/SA14142m^ could block lethal GV Muar infection in mice (Figure 6). It is generally accepted that neutralizing antibodies with a PRNT_50_ of 1:10 or greater are sufficient to protect against the onset of JE [42]. However, the PRNT_50_ of sera from three mice inoculated with 1 × 10^5^ PFU of the mutant viruses against Muar were lower than 1:10 (Table 2). All mutant virus-inoculated mouse sera, including <1:10 of PRNT_50_ against Muar, were positive for antibodies against Muar in IFA (Table 2), indicating that all of the mice were immunized with the mutant viruses. Cellular immunity to non-structural proteins of JEV may also be induced in mutant virus-inoculated mice and have a suppressive effect on the pathogenesis caused by Muar infection [43,44,45]. Twenty percent of mice inoculated with 1 × 10^4^ PFU E^M41/SA14142m^ in vitro and in of E^M41/SA14142m^ or E^XZ/SA14142m^ died; however, 10% of mice in the E^M41/SA14142m^ group and 80% of mice in E^XZ/SA14142m^ group died when 1 × 10^3^ PFU of the mutant viruses were inoculated (Figure 7B). As mentioned above, the growth ability of E^XZ/SA14142m^ was lower than that of vivo, suggesting that E^XZ/SA14142m^ does not amplify sufficiently to induce humoral and cellular immunity in mice infected with the virus at lower doses. It might be possible to recover the growth capacity and immunogenicity of E^XZ/SA14142m^ by reducing the number of amino acid substitutions. Previous reports suggest that four amino acid residues at positions 107, 138, 176, and 244 of the 10 substitution sites play essential roles in the attenuation of neurovirulence and neuroinvasiveness of the SA 14 strain, but at least two at positions 177 and 264 seem to be dispensable for such attenuation [33,34,36,41]. A recombinant JEV with a minimum of four amino acid substitutions in the GV E protein may be a safe and highly efficacious live vaccine against GV JEV infection. The reverse genetics of GV JEV have already been established [18,46]. This method could be used to generate mutant GV JEV with the same amino acid substitutions in the E protein, and the viruses may represent highly proliferative but safe and effective live vaccine candidates. However, we previously showed that the growth of Muar is deficient in mouse neuroblastoma cells [19,22], indicating that the GV-based mutants would have low replicative potential in mouse-derived cells. Increasing the growth capacity of GVs by introducing new mutations might be required for GV-based live attenuated vaccine development. Our previous data showed that Muar could grow efficiently in human neuroblastoma-derived IMR-32 cells [21]. Thus the low growth ability of GV JEV in mouse-derived cells may not be critical for the development of live attenuated recombinant GV JE vaccines in humans.

Most amino acid residues in the 10 sites we introduced specific amino acid substitutions in this study are also conserved in JE serocomplex flaviviruses, the West Nile virus, Usutu virus, and Murray Valley encephalitis virus (Table S2). A previous report indicated that introducing all 10 substitutions into the attenuated West Nile virus strain W1806 resulted in defective virus propagation in mammalian cells [47]. ChimeriVax-WN, which has the prM-E region of West Nile virus in the live attenuated yellow fever vaccine strain 17D backbone, has three amino acid substitutions L107F, A316V (A315V in JEV), and K440R (K439R in JEV), and the mutations are critical for the reduction in neurovirulence of the chimeric virus [48]. However, a single E138K mutation in the E protein of West Nile virus does not attenuate its virulence [49]. Thus, the “multiple substitution method” could apply to the development of live attenuated vaccines for JE serocomplex flavivirus infections, but would also require careful selection of the positions introducing amino acid substitutions.

In this study, we used a mouse infection model to evaluate the protection and immunogenicity potency of the mutant viruses. However, a more comprehensive analysis using mice and non-human primates is needed to assess the safety and efficacy of the mutant virus against JEV infection before considering its application to humans.

## Figures and Tables

**Table 1:** Neutralization titers (PRNT_50_) of sera from mice infected with recombinant JEVs against GI, GV, and recombinant JEVs ^1^.

Mouse	Mouse No.	PRNT_50_ against:				
		Muar (GV)	Mie/41/2002 (GI)	E^XZ0934^	E^XZ/SA14142m^	E^M41/SA14142m^
Mie/41/2002-inoculated group	1	40	160	80	NT ^2^	NT
	2	80	320	40		
	3	160	620	10		
	4	320	640	40		
	5	80	640	20		
	6	160	1280	20		
	7	160	640	20		
	8	160	320	40		
	median	160	480	30		
E^M41/SA14142m^-inoculated group	1	<10	40	<10	NT	160
	2	20	40	10		320
	3	<10	80	10		320
	4	20	160	20		640
	5	40	160	20		320
	6	10	40	<10		80
	7	20	40	<10		320
	8	10	160	10		1280
	9	20	80	<10		320
	10	40	160	10		1280
	median	20	80	10		320
E^XZ0934^-inoculated group	1	640	40	640	NT	NT
	2	320	80	320		
	3	640	160	640		
	median	640	80	640		
E^XZ/SA14142m^-inoculated group	1	80	<10	160	160	NT
	2	40	<10	20	80	
	3	<10	<10	<10	<10	
	4	20	<10	10	40	
	5	20	<10	10	20	
	6	10	<10	20	20	
	7	20	<10	10	10	
	8	80	<10	40	40	
	9	20	<10	10	20	
	10	10	<10	<10	40	
	median	20	<10	15	30	

^1^ Serum was recovered from mice 3 weeks after inoculation of the viruses and then used for PRNT. ^2^ NT, not tested.

**Table 2:** Neutralization titers (PRNT_50_) and results of indirect immunofluorescent analysis (IFA) of sera from mice inoculated with one or two doses of recombinant JEVs ^1^.

Mouse	Mouse No.	PRNT_50_ against Muar	IFA (Serum Dilution Rate) ^2^	
			×20	×200
E^M41/SA14142m^ 1 dose group	1	20	+	±
	2	40	+	+
	3	<10	+	±
	4	80	+	+
	5	20	+	+
	6	40	+	−
	7	20	+	−
	8	20	+	−
	9	40	+	+
	10	80	+	+
	median	40		
E^M41/SA14142m^ 2 doses group	1	40	+	+
	2	40	+	−
	3	160	+	±
	4	20	+	+
	5	40	+	+
	6	40	+	+
	7	40	+	−
	8	40	+	+
	9	40	+	+
	10	10	+	+
	median	40		
E^XZ/SA14142m^ 1 dose group	1	<10	+	−
	2	160	+	±
	3	10	+	−
	4	40	+	+
	5	20	+	±
	6	20	+	−
	7	10	+	−
	8	20	+	±
	9	10	+	+
	10	20	+	+
	median	20		
E^XZ/SA14142m^ 2 doses group	1	320	+	+
	2	160	+	+
	3	80	+	±
	4	<10	+	+
	5	320	+	+
	6	320	+	+
	7	320	+	+
	8	320	+	+
	9	160	+	+
	10	40	+	+
	median	320		

^1^ Serum was recovered from mice 5 weeks after the initial inoculation of viruses and then used for PRNT against GV Muar strain and for IFA to stain Muar-infected Vero cells. ^2^ +, positive; −, negative; ±, uncertain.

## Data Availability

The data presented in this study are available on request from the corresponding author.

## Supplementary Materials

The following are available online at https://www.mdpi.com/article/10.3390/vaccines9101077/s1, Table S1: Comparison of the amino acid residues on 10 sites in JEV E protein. Table S2: Comparison of the amino acid residues at 10 sites in E protein of JEV, West Nile virus, Usutu virus, Murray valley encephalitis virus, and St. Louis encephalitis virus. Figure S1: Alignment of the nucleotide sequences of E region of recombinant JEV strains. Figure S2: Body weight of mice inoculated with recombinant JEV strains as shown in Figure 3A. Figure S3: Body weight of mice inoculated with recombinant JEV strains as shown in Figure 6C. Figure S4: Body weight of mice inoculated with recombinant JEV strains as shown in Figure 7B. Figure S5: Comparison of amino acid sequences of E protein of GV JEV Muar and XZ0934 strains.
