# Supplementary material for: Immunogenicity and Protective Ability of Genotype I-Based Recombinant Japanese Encephalitis Virus (JEV) with Attenuation Mutations in E Protein against Genotype V JEV
Source: Vaccines (Basel). 2021 Sep 25;9(10):1077. doi: 10.3390/vaccines9101077 (PMC8538582; doi:10.3390/vaccines9101077)
Supplement: Supplementary file 1 [file vaccines-09-01077-s001.zip › vaccines-1378159-supplementary.pdf]

Table S1

Table S1. Comparison of the amino acid residues at 10 sites in JEV E protein.

Strain (genotype, accession no.)	Amino acid position in E protein									
	107	138	176	177	244	264	279	315	439	447
SA 14-14-2 (GIII, AF315119)	F	K	V	A	G	H	M	V	R	D
SA 14 (GIII, U04522)	L	E	I	T	E	Q	K	A	K	G
Mie/41/2002 (GI, AB241119)	L	E	I	T	E	Q	K	A	K	G
Mie/51/2006 (GI, AB698905)	L	E	I	T	E	Q	K	A	K	G
Hiroshima/46/1998 (GI, AB174873)	L	E	I	T	E	Q	K	A	K	G
Tokyo602/2005 (GI, AB698908)	L	E	I	T	E	Q	K	A	K	G
JaNAr0102.2002.Mo (GI, AY377577)	L	E	I	T	E	Q	K	A	K	G
XJ69 (GI, EU880214)	L	E	I	T	E	Q	K	A	K	G
GZ56/2006 (GI, HM366552)	L	E	I	T	E	Q	K	A	K	G
CH2010.3 (GI, JF499821)	L	E	I	T	E	Q	K	A	K	G
K91P55 (GI, U34928)	L	E	I	T	E	Q	K	A	K	G
K94P05 (GI, U34929)	L	E	I	T	Q	Q	K	A	K	G
2372Thai79 (GI, U70401)	L	K	I	T	E	Q	K	A	K	G
B2239Thai84 (GI, U70391)	L	E	I	T	E	Q	K	A	K	G
VN105.Mo (GI, AY376468)	L	E	I	T	E	Q	K	A	K	G
FU (GII, AF217620)	L	E	I	T	E	Q	K	A	K	G
Beijing-1 (GIII, AB920348)	L	E	I	T	E	Q	K	A	K	G
Nakayama (GIII, AB920347)	L	E	I	T	E	Q	K	A	K	G
JaTH160 (GIII, AB269326)	L	E	I	T	E	Q	K	A	K	G
JaTAn1/90 (GIII, AB551991)	L	E	I	T	E	Q	K	A	K	G
GZ042 (GIII, JN381857)	L	E	I	T	E	Q	K	A	K	G
JKT6468 (GIV, AY184212)	L	E	I	T	E	Q	K	A	K	G
Muar (GV, HM596272)	L	E	I	T	E	Q	K	A	K	G
XZ0934 (GV, JF915894)	L	E	I	T	E	Q	K	A	K	G
10-1827 (GV, JN587258)	L	E	I	T	E	Q	K	A	K	G
K15P38 (GV, MF526903)	L	E	I	T	E	Q	K	A	K	G

Table S2

Table S2. Comparison of the amino acid residues at 10 sites in E protein of JEV, West Nile virus, Usutu virus, Murray valley encephalitis virus, and St. Louis encephalitis virus.

Strains (Accession no.)	Amino acid position in E protein*									
	107	138	176	177	244	264	279	315	439	447
SA-14-14-2 (AF315119)	F	K	V	A	G	H	M	V	R	D
SA-14 (U04522)	**L**	**E**	**I**	**T**	**E**	**Q**	**K**	**A**	**K**	**G**
West Nile virus (DQ211652)	**L**	**E**	Y	**T**	**E**	**Q**	**K**	**A**	**K**	**G**
Usutu virus (NC_006551)	**L**	**E**	**I**	**T**	**E**	**Q**	**K**	**A**	**K**	**G**
Murray valley encephalitis virus (NC_000943)	**L**	**E**	**I**	**T**	**E**	**Q**	**K**	**A**	**K**	**G**
St. Louis encephalitis virus (NC_007580)	**L**	**E**	F	**T**	**E**	T	T	T	**K**	**G**

*Amino acid position number in JEV E protein.

Figure S1

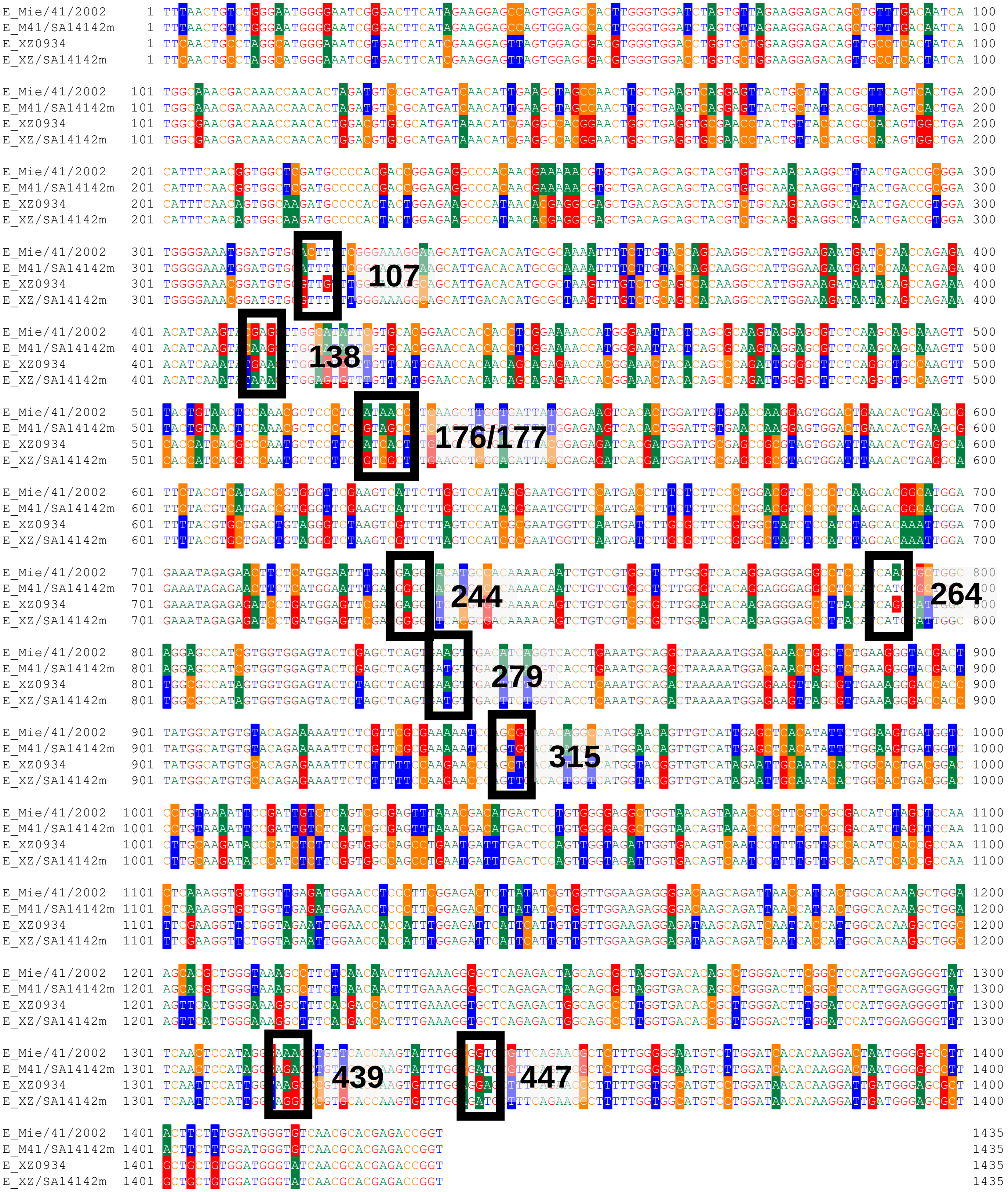

Figure S1. Alignment of the nucleotide sequences of E region of recombinant JEV strains. Boxes indicate the mutation sites introduced.

Figure S2

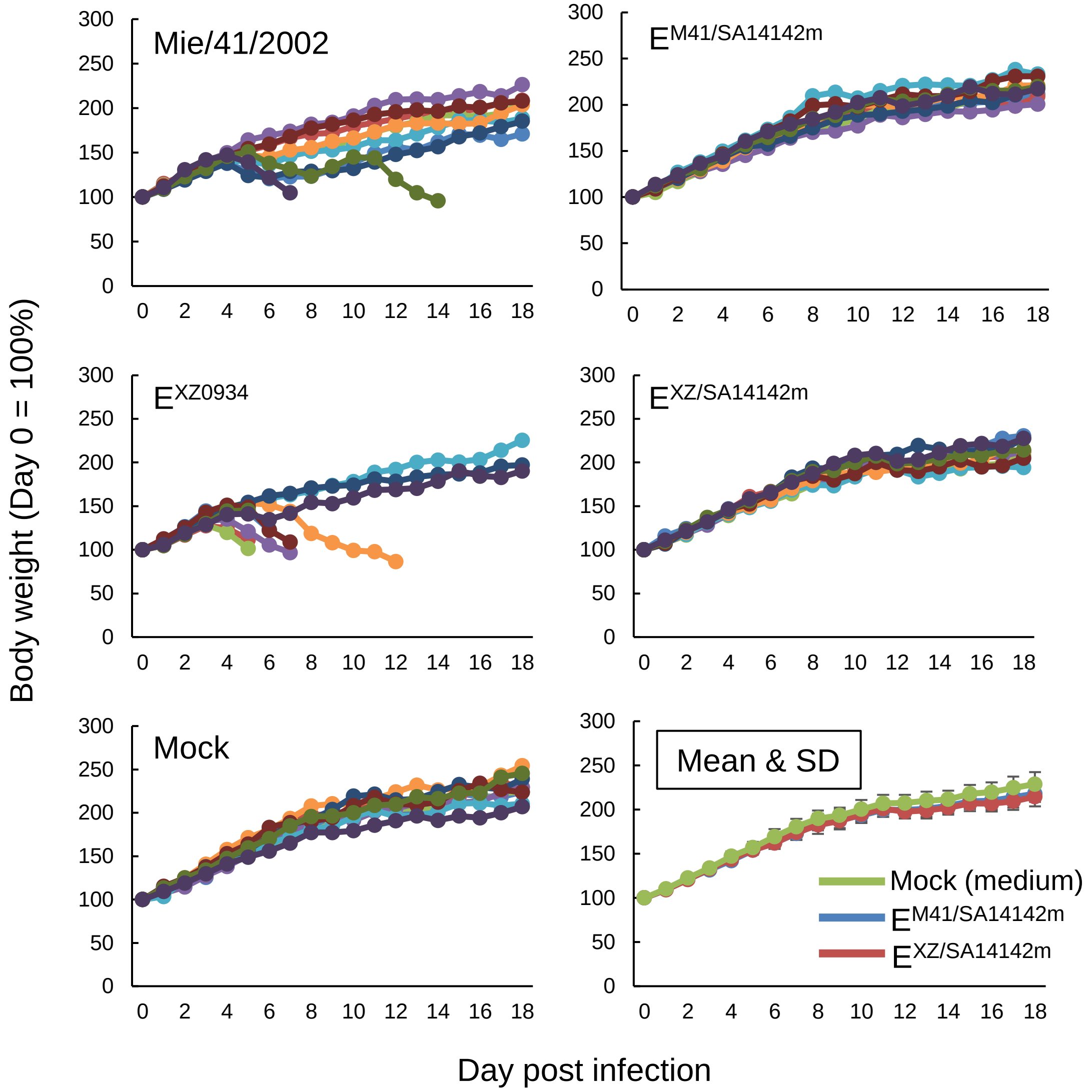

Figure S2. Body weight of mice inoculated with recombinant JEV strains as shown in Figure 3A.

Figure S3

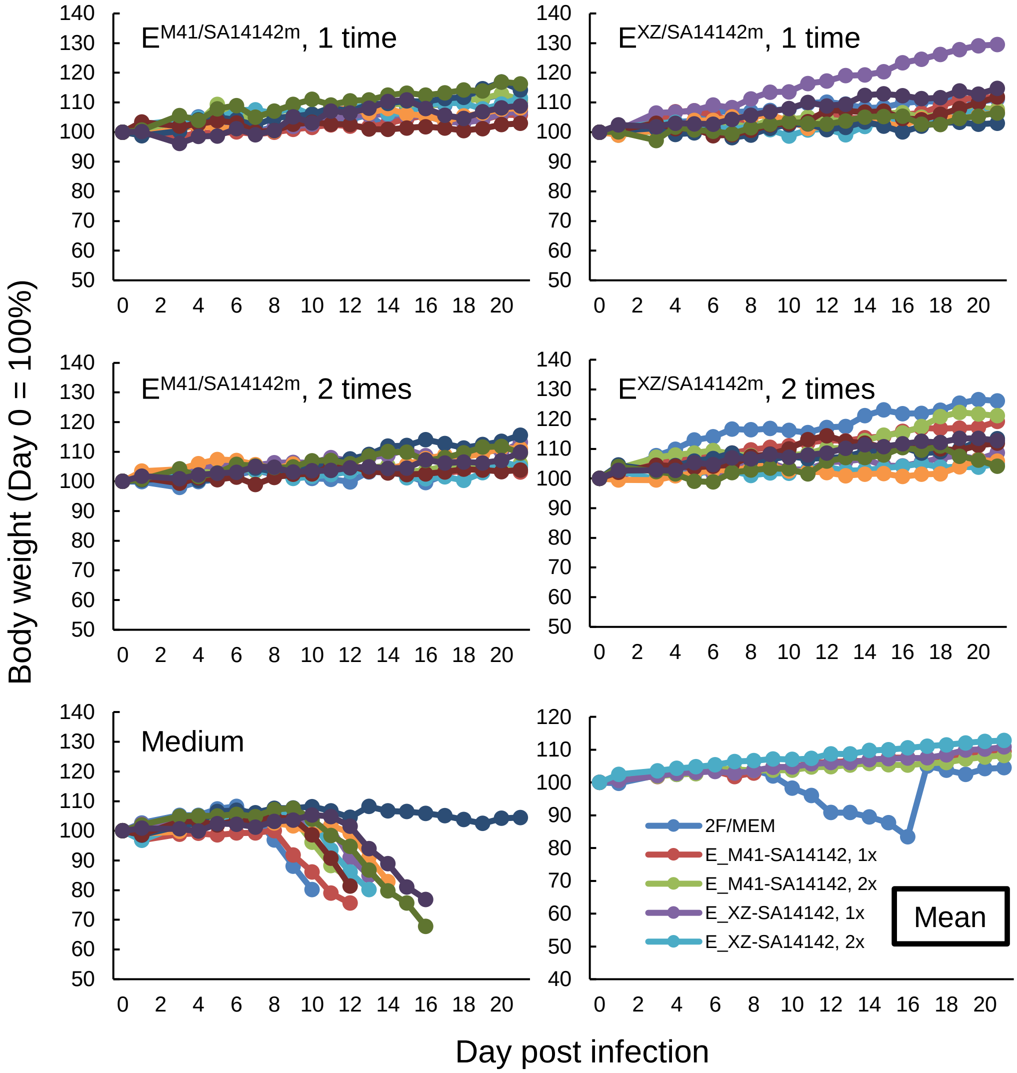

Figure S3. Body weight of mice inoculated with recombinant JEV strains as shown in Figure 6C.

Figure S4

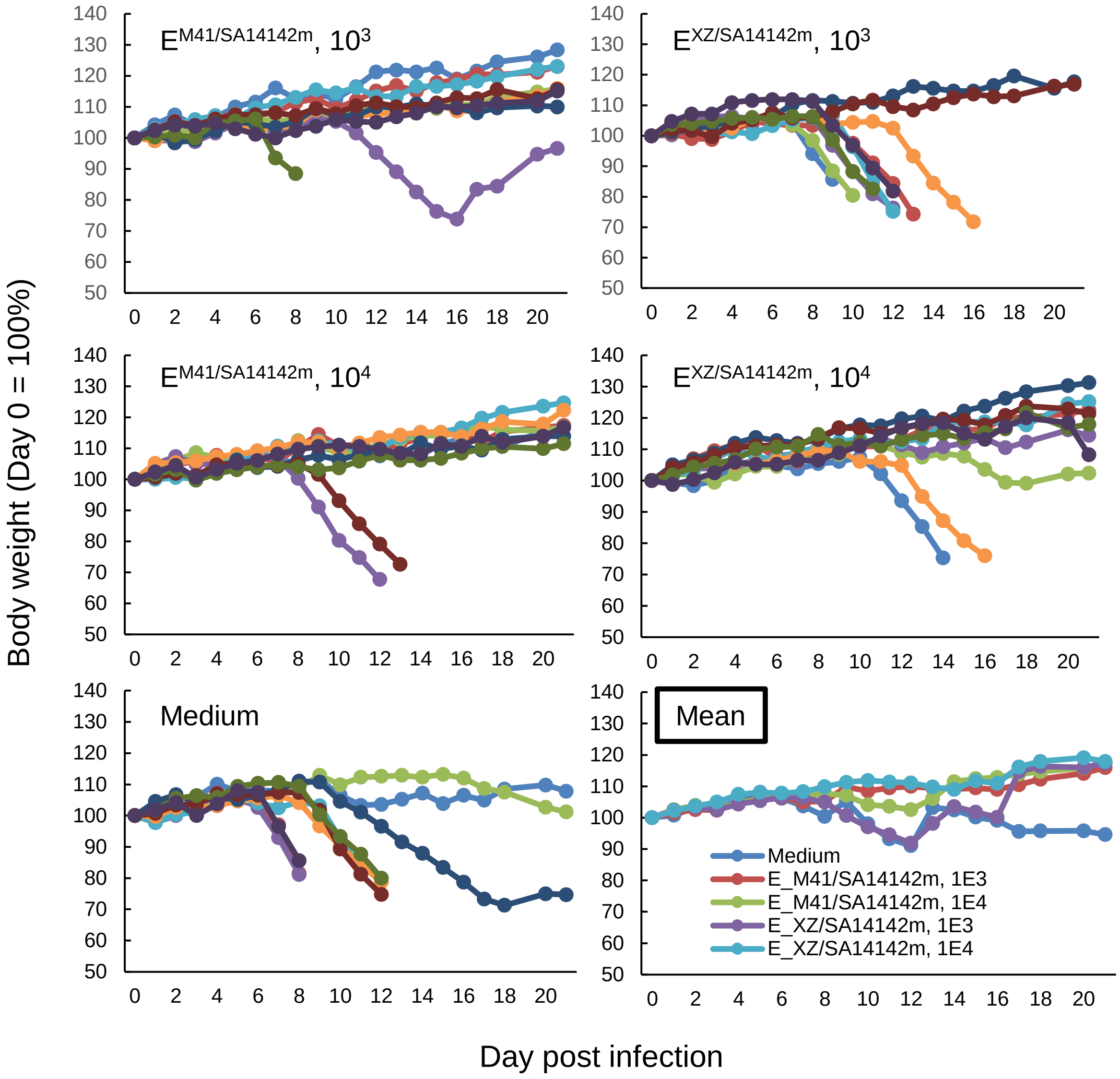

Figure S4. Body weight of mice inoculated with recombinant JEV strains as shown in Figure 7B.

Figure S5

```
JE.E(V).Muar.pep      1 FNCLGMGNRDFIEGVSGATWVDLVLEGDSCLTIMANDKPTLDVRMINIEA 50
JE.E(V).XZ0934.pep    1 FNCLGMGNRDFIEGVSGATWVDLVLEGDSCLTIMANDKPTLDVRMINIEA 50

JE.E(V).Muar.pep     51 TQLAEVRTYCYHATVADISTVARCPTTGEAHNTRRADSSYVCKQGYTDRG 100
JE.E(V).XZ0934.pep   51 TELAEVRTYCYHATVADISTVARCPTTGEAHNTRRADSSYVCKQGYTDRG 100

JE.E(V).Muar.pep    101 WGNGCGLFGKGSIDTCAKFVCSHKAIGKIIQPENIKYEVGVFVHGTTTAE 150
JE.E(V).XZ0934.pep  101 WGNGCGLFGKGSIDTCAKFVCSHKAIGKIIQPENIKYEVGVFVHGTTTAE 150

JE.E(V).Muar.pep    151 NHGNYSAQIGASQAAKFTITPNAPSITLKLGDYGEVTMDCEPRSGFNTEA 200
JE.E(V).XZ0934.pep  151 NHGNYTAQIGASQAAKFTITPNAPSITLKLGDYGEITMDCEPRSGFNTEA 200

JE.E(V).Muar.pep    201 FYVLTVGTKSFLVHREWFNDLALPWLSPSSTNWRNREILLEFEEAHATKQ 250
JE.E(V).XZ0934.pep  201 FYVLTVGSKSFLVHREWFNDLALPWLSPSSTNWRNREILMEFEEAHATKQ 250

JE.E(V).Muar.pep    251 SVVALGSQEGALHQALAGAIVVEYSSSVKLTSGHLKCRLKMDKLALKGTT 300
JE.E(V).XZ0934.pep  251 SVVALGSQEGALHQALAGAIVVEYSSSVKLTSGHLKCRLKMEKLALKGTT 300

JE.E(V).Muar.pep    301 YGMCTEKFSFSKNPADTGHGTVVIELQYTGTDGPCKIPISSVASLNDLTP 350
JE.E(V).XZ0934.pep  301 YGMCTEKFSFSKNPADTGHGTVVIELQYTGTDGPCKIPISSVASLNDLTP 350

JE.E(V).Muar.pep    351 VGRLVTVNPFVATSTANSKVLVELEPPFGDSFIVVGRGDKQINHHWHKAG 400
JE.E(V).XZ0934.pep  351 VGRLVTVNPFVATSTANSKVLVELEPPFGDSFIVVGRGDKQINHHWHKAG 400

JE.E(V).Muar.pep    401 SSLGKAFTTTLKGAQRLAALGDTAWDFGSIGGVFNSIGKAVHQVFGGAFR 450
JE.E(V).XZ0934.pep  401 SSLGKAFTTTLKGAQRLAALGDTAWDFGSIGGVFNSIGKAVHQVFGGAFR 450

JE.E(V).Muar.pep    451 TLFGGMSWITQGLMGALLLWMGINARDRSIALAFLATGGVLLFLATNVHA 500
JE.E(V).XZ0934.pep  451 TLFGGMSWITQGLMGALLLWMGINARDRSIALAFLATGGVLLFLATNVHA 500
```

Figure S5. Comparison of amino acid sequences of E protein of GV JEV Muar and XZ0934 strains.
